# Initial Experiences of Laparoscopic Nephrectomy in a Tertiary Oncology Center: An Analysis of 142 Cases

**DOI:** 10.7759/cureus.59382

**Published:** 2024-04-30

**Authors:** Mehmet Duvarcı, Oğuzhan Ceylan, Murat Beyatlı, Tuncel Uzel, Erdem Öztürk, Nurullah Hamidi, Halil Başar

**Affiliations:** 1 Urology, Dr. Abdurrahman Yurtaslan Oncology Training and Research Hospital, Ankara, TUR; 2 Urology, Umraniye Training and Research Hospital, Sakarya, TUR

**Keywords:** laparoscopic surgery, nephrectomy, laparoscopy, kidney tumor, laparoscopic nephrectomy

## Abstract

Introduction: Kidney tumors have an important place among urological malignancies. The increased utilization of imaging methods has led to a rise in renal cell carcinoma (RCC) diagnoses, albeit with declining mortality rates, particularly in developed countries. Radical nephrectomy remains the gold standard treatment. The aim of this study was to share a tertiary oncology hospital's initial experiences with laparoscopic nephrectomy.

Materials and methods: This retrospective study analyzes data from patients who underwent laparoscopic nephrectomy, focusing on demographic characteristics, tumor features, and operative outcomes. Information regarding age, gender, tumor size, operative details, and pathology results was collected and analyzed.

Results: One hundred forty-two patients were included in the study; 69 (48.60%) were female and 73 (51.40%) were male. The mean age of the patients was 57.11 ± 12.6 years, with tumors primarily located on the left kidney (52.80%). The mean tumor size was 53.01 ± 24.01 mm. Intraoperative complications included the need for conversion to open surgery in five cases and vascular, pneumothorax, or duodenal injuries in a subset of patients. However, postoperative complications, such as sepsis or mortality, were not observed.

Discussion: Despite an initial learning curve associated with longer operation times, laparoscopic techniques offer benefits, including reduced blood loss, faster recovery, and improved cosmetic outcomes. Histologically, clear cell RCC was the most common tumor type encountered. This study underscores the safety and efficacy of laparoscopic radical nephrectomy, advocating for its widespread adoption while emphasizing the importance of surgeon experience and patient selection in optimizing outcomes.

## Introduction

Kidney tumors are the third most common urological tumor and constitute approximately 2-3% of annual cancer cases in studies [1]. The increase in the use of imaging methods is one of the main reasons for the increase in the incidence of renal cell carcinoma (RCC). However, there is a decrease in mortality rates, especially in developed countries [2]. Kidney tumors are less symptomatic and when symptomatic, the disease is more advanced. The triad of symptoms is gross hematuria, flank pain, and palpable mass [3].

Radical nephrectomy is one of the methods used for the treatment of kidney tumors. It is classified as open, laparoscopic, and robotically assisted radical nephrectomy. These techniques are known to show similar oncological results. Compared to open surgery, laparoscopic nephrectomy has less hospital stay, earlier return to daily life, and less painkiller use [4]. Although laparoscopic nephrectomy has a longer operation time compared to open nephrectomy in many studies, it is seen that the time decreases as experience increases [5].

In this study, we aimed to analyze the data of patients with laparoscopic nephrectomy performed in our clinic.

## Materials and methods

Patients who underwent laparoscopic nephrectomy in our clinic between February 2015 and August 2022 were included in the study. To maintain patient confidentiality, a primary investigator collected the data without recording any personal information about the patient. The patients included in the study-age, gender, tumor size, operation side, operation information, and pathology results-were examined. SPSS version 25 (IBM Corp., Armonk, NY, USA) was used for statistical analyses. Mean and standard deviation values were used for continuous variables. Frequency and percentages were used for categorical variables.

This study was designed by the Declaration of Helsinki, patient rights regulation, and ethical rules in the Urology Clinic of Health Sciences University Dr. Abdurrahman Yurtlaslan Ankara Oncology Training and Research Hospital and was conducted with the approval of the ethics committee numbered 2022-03/58.

Surgical technique

All patients underwent nephrectomy via a transperitoneal approach. The operation was performed using three or four trocars, according to the body mass index of the patients. Patients were placed in a 60-degree lateral decubitus position after anesthesia. The pneumoperitoneum was created with the Hasson technique [6] in some patients and the Veress needle in others. Two 11 mm and one 5 mm trocars were used. Following the medialization of the colon, the renal artery and vein were dissected following the pedicle. After dissection, a vascular stapler (Endo GIA Universal Straight, Autosuture (45-2.5), Covidien, Dublin, Ireland) was applied to the pedicle. Dissection of the ureter and dissection of the kidney from the surrounding tissues were then completed. The specimen was taken out through a Gibson incision in an endobag.

## Results

A total of 142 patients, 69 (48.60%) of whom were female and 73 (51.40%) were male, were included in our study. Of the patients, 62 had hypertension, 80 did not, and 53 of the patients had diabetes. Demographic and tumor characteristics of patients who underwent laparoscopic radical nephrectomy are given in Table 1. The age of the patients ranged from 15 to 81, with a mean age of 57.11 ± 12.68 years. While 75 (52.80%) of the kidney tumors were on the left side, 67 (47.20%) of them were on the right side. The mean tumor size was found to be 53.01 ± 24.01 mm. Our data are shown in Table 1.

**Table 1:** Patient characteristics. SD: standard deviation, mean ± SD used for continuous variables. N and percentage used for categorical variables.

	Female	Male	Total
Patients	69 (48.60%)	73 (51.40%)	142
Age, mean ± SD (min-max)	57.83 ± 12.84 (15-76)	56.45 ± 12.59 (32-81)	57.11 ± 12.68 (15-81)
Side, N (%)			
Right	31 (46.27%)	36 (53.73%)	67 (47.20%)
Left	38 (50.70%)	37 (49.30%)	75 (52.80%)
Tumor size (mm), mean ± SD (min-max)	53.83 ± 22.63 (14-127)	52.25 ± 24.01 (14-135)	53.01 ± 24.01 (14-135)
Localization, N (%)			
Upper	28 (50.90%)	27 (49.10%)	55 (38.73%)
Mid	18 (42.85%)	24 (57.15%)	42 (29.58%)
Lower	23 (51.10%)	22 (48.90%)	45 (31.69%)
Histological type, N (%)			
Clear cell	37 (42.05%)	51 (57.95%)	88 (62.00%)
Papillary	4 (26.70%)	11 (%73.30)	15 (10.50%)
Chromophobe	7 (63.40%)	4 (36.40%)	11 (7.75%)
Sarcomatoid	5 (83.30%)	1 (16.70%)	6 (4.25%)
Oncocytoma	5 (71.43%)	2 (28.57%)	7 (4.95%)
Angiomyolipoma	9 (90.00%)	1 (10.00%)	10 (7.05%)
Others	2 (40.00%)	3 (60.00%)	5 (3.50%)
Fuhrman grade, N (%)			
1	6 (42.86%)	8 (57.14%)	14 (9.85%)
2	24 (48.00%)	26 (52.00%)	50 (35.20%)
3	14 (35.00%)	26 (65.00%)	40 (28.18%)
4	3 (50.00%)	3 (50.00%)	6 (4.24%)
Unspecified	22 (68.75%)	10 (31.25%)	32 (22.53%)

During the operation, five of our patients needed to switch to open surgery. During the operation, vascular injury occurred in five patients, pneumothorax in two patients, and duodenal injury in one patient. No liver or spleen injury requiring intervention occurred. A transfusion was performed in eight patients in the postoperative period. Three patients were followed up for prolonged ileus. Sepsis, the need for postoperative intensive care, and mortality were not observed in our patients in the postoperative follow-up (Table 2).

**Table 2:** Operational characteristics. SD: standard deviation, mean ± SD used for continuous variables, N and percentage used for categorical variables. HB: hemoglobin, CRE: creatinine.

	Mean ± SD	Minimum	Maximum
Preop HB	13.74 ± 1.76 g/dL	9.1 g/dL	17.7 g/dL
Postop HB	11.92 ± 1.65 g/dL	7.9 g/dL	16.9 g/dL
Preop CRE	0.83 ± 0.20 mg/dL	0.43 mg/dL	1.67 mg/dL
Postop CRE	0.94 ± 0.31 mg/dL	0.45 mg/dL	2.81 mg/dL
Operation time	133.04 ± 38.84 min	75 min	270 min
Hospital stays	4.73 ± 1.67 days	2 days	13 days

## Discussion

Today, laparoscopic radical nephrectomy is performed with three different approaches. These are the transperitoneal, retroperitoneal, and hand-assisted laparoscopic approaches. The most commonly used method is the transperitoneal approach [7]. In our clinic, the transperitoneal approach is preferred. Laparoscopic nephrectomy was first used in 1991 by Clayman et al [8]. Since then, laparoscopy has been increasingly applied in urological surgeries. There was no statistically significant difference between the outcomes of laparoscopic radical nephrectomy and open radical nephrectomy in terms of oncological outcomes [9].

In the literature, the female/male ratio in kidney tumors was 2/3, and in our study, 69 (48.60%) were female and 73 (51.40%) were male [9]. When we look at the literature, it has been emphasized that kidney tumors are mostly seen between the ages of 60 and 70 [10]. While it was pointed out that they are seen at younger ages with increasing imaging methods in recent years, there was also a 15-year-old child among our patients. In our series, the patient's age ranged from 15 to 81 years, with a mean age of 57.11 ± 12.68 years. While the tumor was in the right kidney in 67 of our cases (47.20%), it was in the left kidney in 75 cases (52.80%).

Although laparoscopic radical nephrectomy is commonly performed in tumors of T2 and below, it has been reported that it is also performed in tumors of T2 and above [11,12]. In our series, the mean tumor diameter was 53.01 ± 24.01 mm (14-135 mm). Since it involves a learning curve at the beginning, long operation times are seen. While the mean operation time was 181 minutes in Demir et al.'s 32-case laparoscopic radical nephrectomy series [13], the mean operation time was 133.04 ± 38.84 min (75-270 min) in our series. In our series, it was observed that the first cases ended in a longer time. In many series, laparoscopic radical nephrectomy causes less blood loss compared to open surgery, patients need fewer painkillers, intestinal activities return more quickly, hospitalization times are shorter, wound infection rates are less, and healing is faster and faster. Cosmetics have been shown to be better [14-16]. Similar results are observed in our cases.

According to the literature, clear cell RCC accounts for 70-80% of all RCCs, 10-15% for papillary RCC, and 3-5% for chromophobic RCC [17-19]. In our cases, clear cell RCC in 88 (62%) materials, papillary RCC in 15 (10.5%) materials, RCC with chromophobe cells in 11 (7.75%) materials, oncocytoma in 7 (4.95%) materials, sarcomatoid RCC was reported in 6 (4.25%) materials, and angiomyolipoma was reported in 10 (7.05%) materials. Of the patients who operated for tumors, 10 (7.05%) had angiomyolipoma and 7 (4.25%) had oncocytoma, and it should be kept in mind that benign pathologies may also be seen as a result of this operation.

Although it was predicted that prolonged ileus may develop in operations performed with the transperitoneal method in the early stages of surgical practice, it was observed that there was no difference in postoperative ileus in a prospective study comparing the two transperitoneal and retroperitoneal techniques [20]. In our study, prolonged ileus was observed in only three patients. Which method to choose depends on the experience of the surgeon. The generally applied method is the transperitoneal approach. In the first series of patients on this subject, high rates of exposure and complications were observed. In a multicenter study, Desai et al. [20] reported the complication rate as 16% and conversion to open surgery as 5% in laparoscopic nephrectomies; Kural et al. reported 4% and 1.7% [21], in their first 100 cases, reported these rates as 3% and 5%. Although high complication and conversion rates were observed in the first patient series, major complication rates were reported as 3.6% and 5.4%, respectively, and conversion to open surgery was reported as 1.1% and 1.2% in the renal surgery series of 350 and 1311 cases by Soulie et al. and Deziel et al., and they were lower [22,23]. In our series, two patients needed to open due to adhesions thought to be caused by previous surgery, two cases required opening due to uncontrollable bleeding, and one patient needed to open due to duodenal injury. In our series, the mean hospital stay was 4.73 ± 1.67 days (two to 13 days), and drain removal was 2.3 days (two to four days). All of our patients were mobilized on the postoperative first day and started to take oral.

The limitations encountered in our study, subgroup analysis according to years and surgeon's experience have not been performed yet, and our study on this subject continues. Another limitation of ours is that it contains relatively few patients compared to other series in the literature. Oncological results are important for these patient groups, and we have an ongoing study on this subject. The absence of these results in this study is another limitation.

## Conclusions

In conclusion, laparoscopic surgery has shown rapid development in urology as well as in all medical branches in recent years. Laparoscopic radical nephrectomy has become a method that can be easily applied in many clinics in our country with the development of laparoscopic technologies and devices. Laparoscopic radical nephrectomy is a minimally invasive surgical method that can be performed transperitoneally quickly and effectively. Laparoscopy has now replaced open radical nephrectomy. Knowing the basic principles of laparoscopy, it is possible to reduce morbidity and mortality rates by predicting possible complications that may develop. Good preparation and patient selection are among the factors that should be taken into account, especially in the initial period. We think that laparoscopic radical nephrectomy is a surgical procedure that can be performed safely in terms of its low complication rates, even in the first experience of surgical procedures performed by adhering to the principles of laparoscopic surgery.
